# Preclinical Alzheimer’s disease: how to predict who will decline next year?

**DOI:** 10.1093/braincomms/fcad079

**Published:** 2023-03-21

**Authors:** Bernard J Hanseeuw, Keith A Johnson

**Affiliations:** Department of Neurology, Cliniques Universitaires Saint-Luc, the Institute of Neuroscience, Université catholique de Louvain, 1200 Brussels, Belgium; Department of Radiology, Massachusetts General Hospital, the Gordon Center for Medical Imaging, Harvard Medical School, Boston, MA 02114, USA; Department of Radiology, Massachusetts General Hospital, the Gordon Center for Medical Imaging, Harvard Medical School, Boston, MA 02114, USA; Center for Alzheimer Research and Treatment, Department of Neurology, Brigham and Women’s Hospital, Harvard Medical School, Boston, MA 02115, USA

## Abstract

This scientific commentary refers to ‘Medial temporal tau predicts memory decline in cognitively unimpaired elderly’, by Kwan *et al*. (https://doi.org/10.1093/braincomms/fcac325).

**This scientific commentary refers to “Medial temporal tau predicts memory decline in cognitively unimpaired elderly”, by Kwan** ***et al.*** **(https://doi.org/10.1093/braincomms/fcac325)**.

In 2018, the National Institute on Aging and Alzheimer’s Association (NIA-AA) proposed a research framework shifting the definition of Alzheimer’s disease in living people from a clinical to a biological construct.^1^ Yet in 2023, cognitive decline remains paramount to both diagnose Alzheimer’s disease clinically^2^ and evaluate the efficacy of therapeutic trials.^3^ Therefore, observing cognitive decline over short follow-up periods, e.g. in a single year, is critically important to run cost-effective clinical trials. Nevertheless, cognitive decline is more subtle and difficult to detect in clinically normal persons, with the association between elevated brain amyloid and subsequent cognitive decline detectable with current methods after three years.^4^ Thus, identifying clinically unimpaired individuals at-risk of fast cognitive decline would likely accelerate the search for an effective Alzheimer’s disease prevention therapy.

Over the past decade, several research works have attempted to further characterize older adults with elevated brain amyloid. Early work used non-specific neurodegenerative biomarkers, such as brain volume or cerebral glucose metabolism,^5^ whereas more recent work used new radiotracers to evaluate the deposition of tau pathology in neocortex.^6^ Because the proportion of older adults with elevated tau in neocortex is typically low (5–10%), scientists have searched for the origin of tau pathology in the entorhinal cortex.^7^ A large multi-centre study of preclinical Alzheimer’s disease recently demonstrated that the presence of tau pathology in the medial temporal lobe (MTL) was associated with a six-fold increased risk of progression to mild cognitive impairment after a 39-month follow-up (A+T_MTL_+ versus A+T−, Fig. 1).^8^ However, these previous studies mostly used ^F18^Flortaucipir as a radiotracer, which is not optimal to evaluate tau pathology in the hippocampus, because of off-target binding from the adjacent choroid plexus.^9^

**Figure 1:**
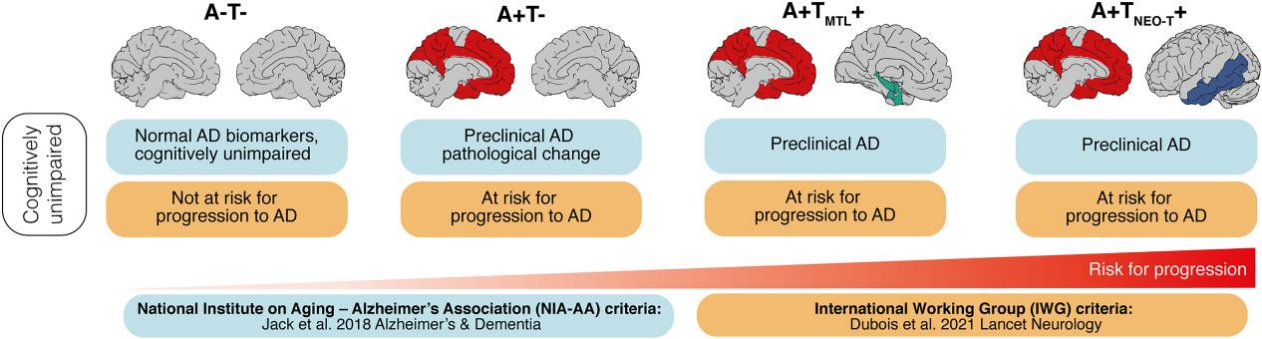
**NIA-AA and International Working Group (IWG) criteria.** Despite differences in the nomenclature of cognitively unimpaired persons defining Alzheimer’s disease biologically (NIA-AA) or clinically (IWG), both classifications recognize the increased risk for progression to symptomatic Alzheimer’s disease when amyloid and tau biomarkers are both positive. Measuring tau in the medial temporal lobe (MTL) may be associated with an earlier detection whereas tau in the neocortex (NEO) may be associated with an increased risk of cognitive decline. Reproduced from: Ossenkoppele, R., Pichet Binette, A., Groot, C. *et al*. Amyloid and tau PET-positive cognitively unimpaired individuals are at high risk for future cognitive decline. Nat Med 28, 2381–2387 (2022)^8^ with the kind permission of Rik Ossenkoppele, Alexa Pichet Binette and Oskar Hansson. Copyright 2022 The author(s): http://creativecommons.org/licenses/by/4.0/

According to neuropathological reports, tau pathology affects the hippocampus in Braak stage 2, together with the amygdala; i.e. tau affects these MTL structures in a greater proportion of the population than the inferior temporal cortex or the rest of the neocortex (Braak stage ≥3). Because it reflects a greater extent of the pathology than entorhinal cortex (Braak stage 1), hippocampal or amygdala tau might be predictive of faster cognitive decline than entorhinal tau. In their recent article in *Brain Communications*, Kwan *et al*.^10^ used ^F18^MK6240 to quantify tau pathology, a radiotracer that is less affected by the choroid plexus off-target binding. They measured tau pathology in a Braak 1–2 region-of-interest, including both the entorhinal cortex and the hippocampus, and observed that twenty of 111 clinically unimpaired participants (18%) had high MK2640 binding in this region. Most importantly, after twelve months of follow-up, significantly faster memory decline was observed in the ten individuals with A+T+ than in the thirteen individuals with A+T− or the eight individuals with A−T+. Thus, this study demonstrates that cognitive decline is observable in a period as short as a year in clinically unimpaired older adults with both amyloid and tau pathologies, when tau is measured in the MTL.

Future studies should specifically compare the sensitivity and specificity of tau-PET positivity in the entorhinal cortex, hippocampus, amygdala and temporal neocortex to predict the onset of mild cognitive impairment over short follow-up periods. In order to adequately plan prevention trials, we will need to know for each region-of-interest the prevalence of tau-PET positivity—which will likely decrease with Braak stages—and the associated risk of cognitive decline—which will likely increase with Braak stages. Different tau-PET regions may be used as inclusion criteria depending on the specific aims of the trial and the drug tested. Future work may also consider using measures of extent or individualized region-of-interest as the stereotypical progression in Braak stages may suffer many exceptions at the individual level. Figure 2 illustrates the case of a 65 years old clinically normal woman with high levels of amyloid and high levels of MTL tau pathology, as observed on these recently acquired ^F18^MK6240 PET images. The images also show high binding in the right occipital lobe, which might further increase the risk of this participant to develop cognitive decline. Without individualized region-of-interest, or measures of the extension of tau pathology, it will be difficult to group this participant with other cases following the Braak stages progression.

**Figure 2:**
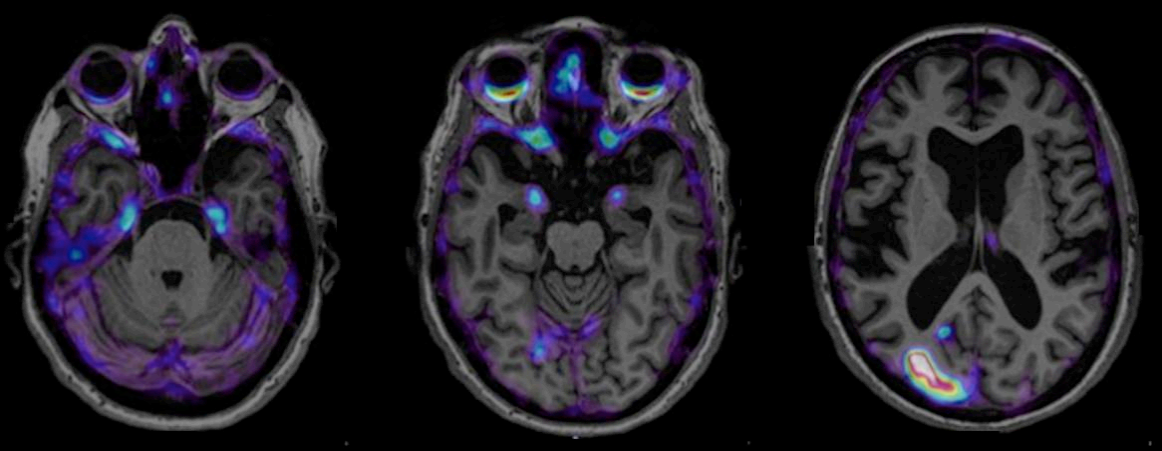
**^F18^MK6240 images of a high-amyloid clinically normal participant.** ^F18^MK6240 images of a high amyloid clinically normal participant aged 65 years old illustrating the regional variability observed with tau-PET, not respecting the stereotypical progression in Braak stages. In this case, high tau-PET signal is observed in the right occipital lobe (Braak stages 5–6) whereas no signal is observed in the inferior temporal cortex (Braak stages 3–4). Images are displayed following the radiological convention. Left: signal clearly visible in the medial temporal lobe; middle: absence of signal in the temporal neocortex; and right: high binding in the right occipital lobe whereas the frontal and parietal lobes do not show any signal.

In conclusion, studies relating cognitive decline to amyloid and tau status, such as this work investigating ^F18^MK6240 signal in the MTL, are much needed in the preparation of clinical trials. Better understanding the clinical meaning of biomarker positivity in cognitively unimpaired and mildly impaired individuals should help us to provide prognosis to these persons and propose them therapeutic interventions to reduce the risk of subsequent cognitive decline.

## Data Availability

The authors have not analysed data from group of participants in this commentary. Additional information about the single case used for illustration can be obtained by sending an email to bhanseeuw@mgh.harvard.edu. Only anonymised information will be communicated after a valid scientific rationale has been provided.
